# *Toxoplasma gondii* inhibits mast cell degranulation by suppressing phospholipase Cγ-mediated Ca^2+^ mobilization

**DOI:** 10.3389/fmicb.2013.00179

**Published:** 2013-07-04

**Authors:** Norah L. Smith, Delbert S. Abi Abdallah, Barbara A. Butcher, Eric Y. Denkers, Barbara Baird, David Holowka

**Affiliations:** ^1^Baker Laboratory, Department of Chemistry and Chemical Biology, Cornell UniversityIthaca, NY, USA; ^2^Department of Microbiology and Immunology, College of Veterinary Medicine, Cornell UniversityIthaca, NY, USA

**Keywords:** IgE, FcεRI, Syk kinase

## Abstract

*Toxoplasma gondii* is well-known to subvert normal immune responses, however, mechanisms are incompletely understood. In particular, its capacity to alter receptor-activated Ca^2+^-mediated signaling processes has not been well-characterized. In initial experiments, we found evidence that *T. gondii* infection inhibits Ca^2+^ responses to fMetLeuPhe in murine macrophages. To further characterize the mechanism of inhibition of Ca^2+^ mobilization by *T. gondii*, we used the well-studied RBL mast cell model to probe the capacity of *T. gondii* to modulate IgE receptor-activated signaling within the first hour of infection. Ca^2+^ mobilization that occurs via IgE/FcεRI signaling leads to granule exocytosis in mast cells. We found that *T. gondii* inhibits antigen-stimulated degranulation in infected cells in a strain-independent manner. Under these conditions, we found that cytoplasmic Ca^2+^ mobilization, particularly antigen-mediated Ca^2+^ release from intracellular stores, is significantly reduced. Furthermore, stimulation-dependent activation of Syk kinase leading to tyrosine phosphorylation and activation of phospholipase Cγ is inhibited by infection. Therefore, we conclude that inhibitory effects of infection are likely due to parasite-mediated inhibition of the tyrosine kinase signaling cascade that results in reduced hydrolysis of phosphatidylinositol 4,5-bisphosphate. Interestingly, inhibition of IgE/FcεRI signaling persists when tachyzoite invasion is arrested via cytochalasin D treatment, suggesting inhibition is mediated by a parasite-derived factor secreted into the cells during the invasion process. Our study provides direct evidence that immune subversion by *T. gondii* is initiated concurrently with invasion.

## Introduction

The apicomplexan *T. gondii* has evolved to be an extremely successful obligate intracellular parasite. It parasitizes a multitude of mammalian and avian species as intermediate hosts. In felines, which serve as the definitive host, sexual reproduction results in shedding of highly infectious oocysts. The Center for Disease Control and Prevention estimates one fifth of the US. human population is latently infected with *T. gondii*. In most hosts, infection is long-lived but asymptomatic. Nevertheless, under certain conditions, such as in immuno-compromised individuals, acute toxoplasmosis poses serious health risks (Dubey, 1998).

*T. gondii* infects host cells through a process of active invasion and establishment of a parasitophorous vacuole that resists fusion with the phago-lysosomal system (Sibley, 2004). One of the probable reasons for the success of *Toxoplasma* as an intracellular pathogen is its development of immuno-modulatory mechanisms to evade and control the host response to infection (Laliberte and Carruthers, 2008; Leng et al., 2009a). *In vivo* infection results in a strong IFN-γ-mediated protective immune response that is necessary for host survival, and, as a result, for parasite survival (Lambert and Barragan, 2010). At the same time, infection actively suppresses production of many pro-inflammatory cytokines (Leng et al., 2009a). Virulence factors such as ROP16 and ROP18 are secreted from parasite rhoptries and act to directly modulate host cell signaling and interfere with host antimicrobial function (Butcher et al., 2005; Saeij et al., 2006; Taylor et al., 2006; Yamamoto et al., 2009, 2011). Ca^2+^ mobilization is a key regulator of many signaling pathways in immune cells, including those that control granule exocytosis, chemotaxis, and gene transcription and expression (Putney, 2009). A recent study demonstrated *Toxoplasma* alteration of Ca^2+^ signaling in neurons during chronic infections (Haroon et al., 2012). Studies of *T. gondii* invasion in the context of a well-established immune model in which Ca^2+^ signaling triggers a rapid response, such as mast cell degranulation, are useful to understand mechanisms by which *Toxoplasma* can modulate Ca^2+^ signaling.

While there is evidence that peritoneal mast cells mount an immune response to *Toxoplasma* infection (Ferreira et al., 2004; Sawesi et al., 2010), mast cells have not been determined to be reservoirs for *T. gondii in vivo*. However, other immune cell types, such as macrophages, dendritic cells, and neutrophils, are known targets of *T. gondii* infection (Bierly et al., 2008; Lambert and Barragan, 2010). In all of these cell types, Ca^2+^-dependent signaling is involved in crucial cellular functions. For example, Ca^2+^-mediated signaling pathways are involved in FcRγ-mediated phagocytosis, inflammation, and nitric oxide synthesis in macrophages (Jongstra-Bilen et al., 2008; Braun et al., 2009; Huang et al., 2012), and C-type lectin signaling in dendritic cells relies on phospholipase C (PLC)γ2 (Xu et al., 2009). In response to N-formyl-L-methionyl-L-leucyl-L-phenylalanine (fMLP), Ca^2+^ mobilization by neutrophils is activated via PLCβ (Andersson et al., 1986; Ferretti et al., 2001).

Mast cells express FcεRI, the high affinity receptor for IgE, and they are primary mediators of the allergic response (Metcalfe et al., 1997). Crosslinking of IgE-FcεRI complexes on the cell surface by oligovalent antigen is the first step in the cascade of signaling events that results in the exocytosis of preformed mediators, such as histamine and serine proteases, with a time course of minutes (Metcalfe et al., 1997). FcεRI belongs to the family of multichain immune recognition receptors (MIRRs) that also include B-cell and T-cell receptors (Cambier, 1995). Signal transduction through FcεRI has been extensively studied by us and others (Holowka et al., 2005; Rivera and Gilfillan, 2006) and involves PLCγ1 and PLCγ2 activation leading to Ca^2+^ mobilization and protein kinase C (PKC) activation, both of which are necessary for stimulated granule exocytosis (Ma and Beaven, 2009; Holowka et al., 2012).

In the present study we demonstrate that Ca^2+^ responses are altered in *Toxoplasma*-infected primary neutrophils, and we utilize the well-established RBL mast cell model system to characterize the mechanism by which *Toxoplasma* rapidly modulates Ca^2+^-mediated immune cell signaling. We find that, within an hour of infection, parasites significantly inhibit antigen-mediated degranulation, primarily by inhibition of inositol 1,4,5-trisphosphate (IP_3_)-dependent Ca^2+^ mobilization. Additional experiments revealed that PLCγ activation by Syk tyrosine kinase is inhibited by *Toxoplasma* infection. Finally, we found that inhibition of degranulation prevails under conditions in which inhibition of actin polymerization prevents parasite invasion. Collectively, these results support a model in which *T. gondii* inhibits FcεRI receptor signaling during invasion by releasing a factor that inhibits Syk mediated activation of PLCγ, and thus interferes with hydrolysis of phosphatidylinositol 4,5-bisphosphate (PIP_2_) to produce the second messengers IP_3_ and diacylglycerol (DAG) important for Ca^2+^ mobilization and degranulation.

## Materials and methods

### Chemicals and reagents

Indo-1-AM and Fluo-4-AM were purchased from Invitrogen Corp. 4-methylumbelliferyl-N-acetyl-β-D-glucosaminide, cytochalasin D, FITC-dextran, and thapsigargin were purchased from Sigma-Aldrich. Unless otherwise noted, all other tissue culture reagents were purchased from Invitrogen, and all other chemicals were purchased from Sigma-Aldrich. Anti-DNP IgE was purified as described previously (Posner et al., 1992). Multivalent antigen, DNP-BSA, was prepared as described previously (Weetall et al., 1993).

### Cells and parasites

#### RBL-2H3 mast cells

RBL-2H3 mast cells were maintained in monolayer culture through weekly passage as described previously (Gidwani et al., 2003). For stimulation, cells were sensitized with 1 μg/ml anti-DNP IgE for 4–24 h.

#### Mouse neutrophils

Female C57BL/6 mice (6–8 weeks of age) were purchased from either The Jackson Laboratory (Bar Harbor, ME) or Taconic Farms (Germantown, NY) and were maintained in the Transgenic Mouse Core Facility at the Cornell University College of Veterinary Medicine, accredited by American Association of Accreditation of Laboratory Animal Care. Mouse neutrophils were isolated by percoll gradient purification as described previously (Abi Abdallah et al., 2011).

The experiments in this study were performed in strict accordance with the recommendations in the Guide for the Care and Use of Laboratory Animals of the National Institutes of Health. The protocols were approved by the Institutional Animal Care and Use Committee at Cornell University (permit number 1995-0057). All efforts were made to minimize animal suffering during the course of these studies.

#### Parasites

RH, PTG, CTG, Veg, and RH-tomato strains of parasites were used in this study. The RH-tomato strain, stably expressing tomato fluorescent protein, was generated by Dr. B. Striepen [University of Georgia; kindly provided by Dr. E. Robey (University of California, Berkeley)]. All tachyzoites were maintained *in vitro* via passage through human foreskin fibroblast cultures in DMEM with FCS (1%), penicillin (100 U/ml) and streptomycin (100 μg/ml) (Fibroblast media). For experiments, tachyzoites were harvested and passed through a 3 μm track-etched membrane filter (Whatman) to remove fibroblast debris. Infections were performed at a multiplicity of infection (MOI) of 10:1 unless otherwise indicated and were synchronized by brief centrifugation (200 × g for 4 min).

### Degranulation

#### β-Hexosaminidase release

Cells were sensitized and plated in triplicate at a density of 5 × 10^5^/well and incubated overnight. The next day, cells were washed with fibroblast media and parasites were introduced as described above. For some experiments, 1 μM cytochalasin D was added during infection to prevent invasion. Following infection, cells were washed three times with buffered saline solution (BSS: 135 mM NaCl, 5 mM KCl, 1 mM MgCl_2_, 1.8 mM CaCl_2_, 5.6 mM glucose, 20 mM HEPES, pH 7.4, 1 mg/ml BSA) and β-hexosaminidase release in response to DNP-BSA was assessed as described previously (Naal et al., 2004).

#### Live cell degranulation imaging

Live cell degranulation imaging experiments were carried out as described previously (Cohen et al., 2012). Briefly, sensitized cells were plated overnight in 35 mm glass bottom dishes (MatTek) in the presence of FITC-dextran (1 mg/ml) and 5-HT serotonin (0.2 mM). The next day, cells were washed with fibroblast media and infected with RH-tomato tachyzoites as described above. Following 1 h of infection, cells were washed three times with BSS. Imaging was conducted at 37°C on a Leica SP5 confocal microscope at an image acquisition rate of 1.7 Hz. Cells were monitored for 1 min prior to addition of multivalent antigen, DNP-BSA (10 ng/ml), then monitored for an additional 9 min.

### Western blotting

Sensitized, adherent cells were infected as described above. Following infection, cells were stimulated for 0–20 min, and whole cell western blotting samples were prepared and blotted as described previously (Young et al., 2005). Samples were run on Tris-glycine gels under reducing conditions. For assessment of phosphorylation, anti-phosphotyrosine (clone 4G10) (Millipore) was used, and pp72 was identified based on molecular weight. To assess sample loading, blots were reprobed with anti-actin (clone ACTN05; Neomarkers). Antibodies against PLCγ1 (Santa Cruz), PLCγ1-pY783, PLCγ2, and PLCγ2-pY1217 (Cell Signaling Technology) were used to assess PLCγ activity. To determine relative intensity, the normalized ratio of phosphorylated band intensity to loading control was calculated, and all values were then normalized as compared to the intensity of the control sample at 20 min post-stimulation.

### Intracellular Ca^2+^ measurements

#### Fluorimetry

Measurement of intracellular Ca^2+^ mobilization in response to antigen (10 ng/ml DNP-BSA), 200 nM thapsigargin or 10 μM fMLP was carried out in tachyzoite infected cells using indo-1 as a Ca^2+^ indicator dye as described previously (Smith et al., 2010). Time-integrated responses were determined as the area under the stimulated time course minus the baseline over 400 s, normalized to the maximal response in Triton X-100 lysed cells (Field et al., 2000).

#### Live cell Ca^2+^ imaging

Single cell Ca^2+^ measurements in infected and control cells were conducted using Fluo-4 AM as described previously (Gadi et al., 2011) on the Leica SP5 confocal system with an image acquisition rate of 0.5 Hz.

Analysis of Ca^2+^ oscillations in individual cells was performed using Matlab software (Mathworks). Briefly, code was written to track the location and average fluorescence intensity of the green channel (Fluo-4) within a circular region of interest (ROI) in the cytoplasm of each cell. These measurements were plotted with respect to time, and the number of oscillations for each ROI, reflected by increases in fluorescence intensity, were enumerated.

### Measurements of phosphoinositide (PIP_2_ and PIP_3_) localization

Cells were sparsely plated (1−3 × 10^5^/ml) on # 1.5 coverslips or in 35 mm glass bottom dishes (MatTek). After overnight culture, cells were transfected with either PH- PLCδ-EGFP (Varnai and Balla, 1998) or PH-Akt-EGFP (Srinivasan et al., 2003) using 2 μg DNA and 8 μl Fugene HD (Roche Diagnostics) in 1 ml OptiMEM for 1 h before addition of 1 ng/ml phorbol 12,13-dibutyrate for 3–5 h to enhance DNA uptake (Gosse et al., 2005). Samples were then washed into full media and cultured for 16–24 h to allow for protein expression.

Transfected cells were infected for 1–2 h with RH-tomato parasites followed by fixation with 4% paraformaldehyde and 0.1% glutaraldehyde in phosphate buffered saline (PBS) for 10 min at room temperature. Excess fixative was quenched by 10 mg/ml BSA in PBS with 0.01% sodium azide. Fixed cells were imaged on a Leica SP2 confocal system. Line scan analysis of equatorial cross sections using ImageJ (NIH) was performed using average fluorescence values to determine the ratio of the PH domain at the plasma membrane to that in the cytoplasm (Smith et al., 2010).

### Statistical analyses

Statistical analysis was performed with Prism software (Graphpad). All bar graphs display mean ± SEM unless otherwise noted. Statistical significance was determined by One-Way ANOVA (Analysis of Variance) followed by Tukey's post test. Level of significance is denoted as follows: ^*^P < 0.05, ^**^P < 0.01, ^***^P < 0.001 and ^****^P < 0.0001.

## Results

### Ca^2+^ responses are reduced in *T. gondii*-infected neutrophils

**Figure 1:**
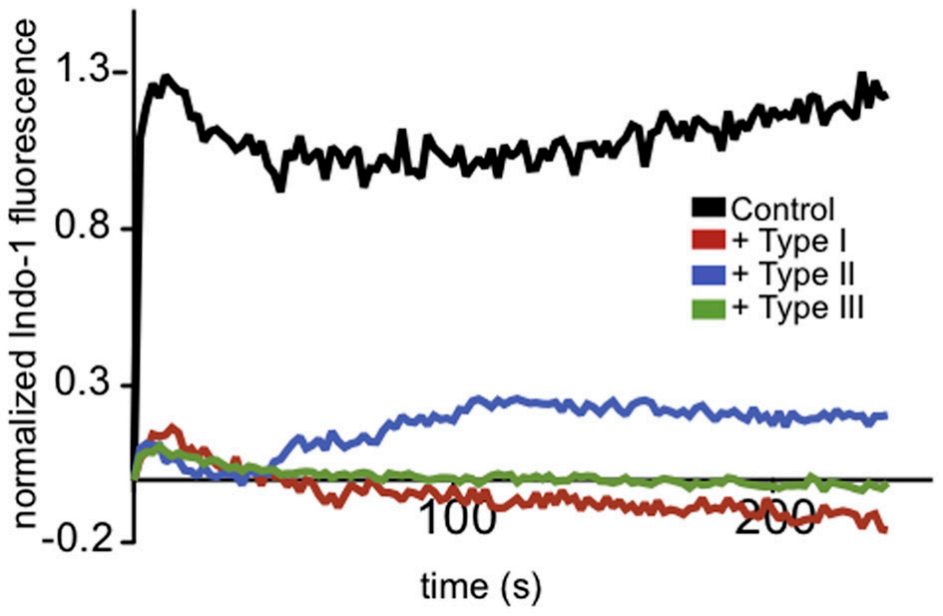
***T. gondii*-infected neutrophils exhibit reduced Ca^2+^ responses to fMLP**. Representative indo-1 fluorescence measurements of Ca^2+^ responses to 10 μM fMLP, added at *t* = 0, in control (black), Type I (red), Type II (blue), or Type III (green) infected cells (MOI 10:1).

*T. gondii* is known to actively modulate signaling in the immune cells it infects (Laliberte and Carruthers, 2008). Ca^2+^ mobilization is central to many aspects of immune signaling, so we examined the effects of parasite infection on neutrophil Ca^2+^ responses to the bacterial chemotactic factor, fMLP. Freshly purified mouse neutrophils were isolated, purified and labeled with indo-1 to monitor Ca^2+^ mobilization stimulated by fMLP following infection by *T. gondii*. As shown in Figure 1, we found that the neutrophil response to fMLP was inhibited by infection with all types of *T. gondii.* These data provided initial evidence that Ca^2+^ signaling in an immune cell type that is an established host target for *T. gondii* is altered by parasite infection.

Live microscopy is a powerful tool to probe the mechanistic aspects of Ca^2+^ responses, but our attempts to use this approach in neutrophils was hampered by their relatively short lifespan *ex vivo* and our findings that neutrophil adhesion on poly-L-lysine-coated glass, necessary for imaging, triggered spontaneous activation, including neutrophil extracellular trap formation (Abi Abdallah, unpublished observations; Abi Abdallah et al., 2012). To overcome these technical limitations, we utilized RBL mast cells as a model system more amenable for investigation of signaling mechanisms important for immune cell-mediated Ca^2+^ responses in the context of *Toxoplasma* infection. Mast cell signaling through the IgE receptor, FcεRI, is a well-studied immune signaling process that occurs on the time scale of minutes. An end result of this signaling is granule fusion and release of histamine, serine proteases and proteoglycans in a process termed degranulation (Blank and Rivera, 2004).

### *T. gondii* infection rapidly inhibits antigen-mediated mast cell degranulation

**Figure 2:**
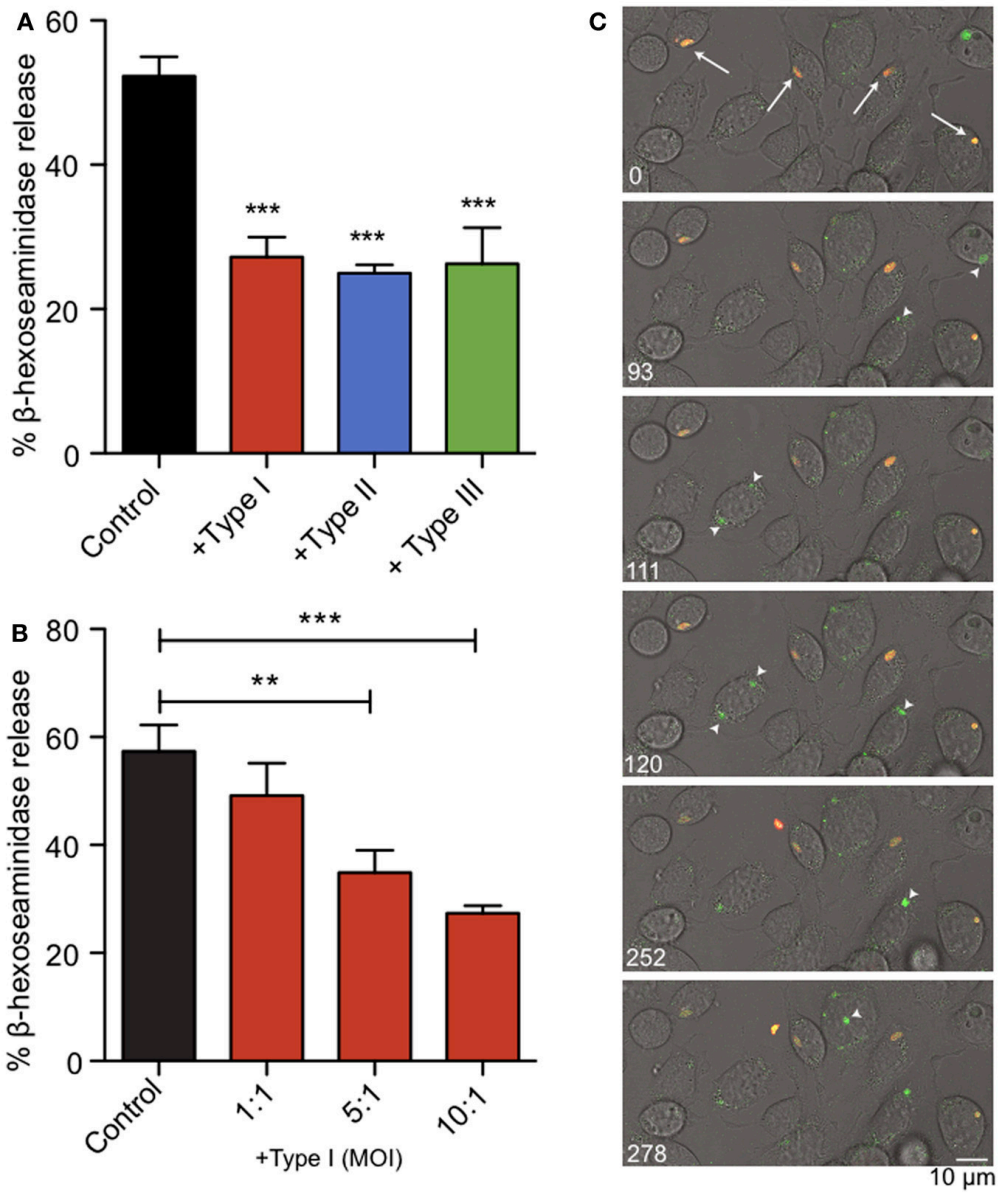
**Acute *T. gondii* infection inhibits antigen-mediated mast cell degranulation. (A)** RBL mast cells were infected with type I (RH, red), type II (PTG, blue) or type III (CTG or VEG, green) tachyzoites for 1 h at an MOI of 10:1 (resulting in 50–70% infection), and degranulation stimulated by antigen (1 ng/ml DNP-BSA) was assessed by β-hexosaminindase release. Results summarize an average of 5–7 experiments, and error bars represent SEM (^**^P < 0.01; ^***^P < 0.001 compared to control uninfected cells). **(B)** A representative experiment showing inhibition of degranulation increases with increasing MOI. **(C)** Individual frames taken from live fluorescence confocal microscopy of degranulation events monitored by FITC-dextran bursts (green) in RBL cells infected with RH-tomato tachyzoites (red) and uninfected RBL cells in the same field. White arrows (timepoint 0) indicate infected cells. Arrowheads highlight degranulation events in uninfected cells (frames taken from supporting movie M1).

We infected RBL-2H3 mast cells with Toxoplasma type I (RH), II (PTG), or III (CTG or VEG) tachyzoites. After 1 h we assessed the capacity of the infected cells to degranulate in response to multivalent antigen, DNP-BSA. In a bulk assay we found that acute *T. gondii* infection reduced degranulation by approximately 50%, irrespective of the genotype of parasite used (Figure 2A). Under these conditions, infection rates were between 50 and 70% and FcεRI receptor expression on the mast cell surface remained unchanged (N.L. Smith, data not shown). This inhibition directly correlated with multiplicity of infection (MOI), suggesting that inhibition is dependent on infection levels (Figure 2B).

We used RH-tomato parasites in live imaging experiments to assess degranulation by FITC-dextran release (Figure 2C, movie M1). In these experiments, FITC-dextran is taken up into the granules of RBL mast cells. FITC fluorescence is pH sensitive and remains quenched in the acidic environment of the granules. Upon stimulation, granules fuse with the plasma membrane, their contents are exposed to a higher pH and local bursts of FITC-dextran fluorescence are detected (Cohen et al., 2012). We found that infected cells are defective in their response to antigen crosslinking and showed delayed exocytosis. Neighboring uninfected cells responded robustly to antigen as seen by bursts of FITC-dextran fluorescence from granules (Figure 2C, arrow heads) and apparent flattening and ruffling of the cells (movie M1). Taken together, these results indicate that *T. gondii* inhibits mast cell degranulation, and this inhibition directly correlates with infection.

### Ca^2+^ mobilization in response to antigen is reduced in *Toxoplasma*-infected RBL-2H3 mast cells

**Figure 3:**
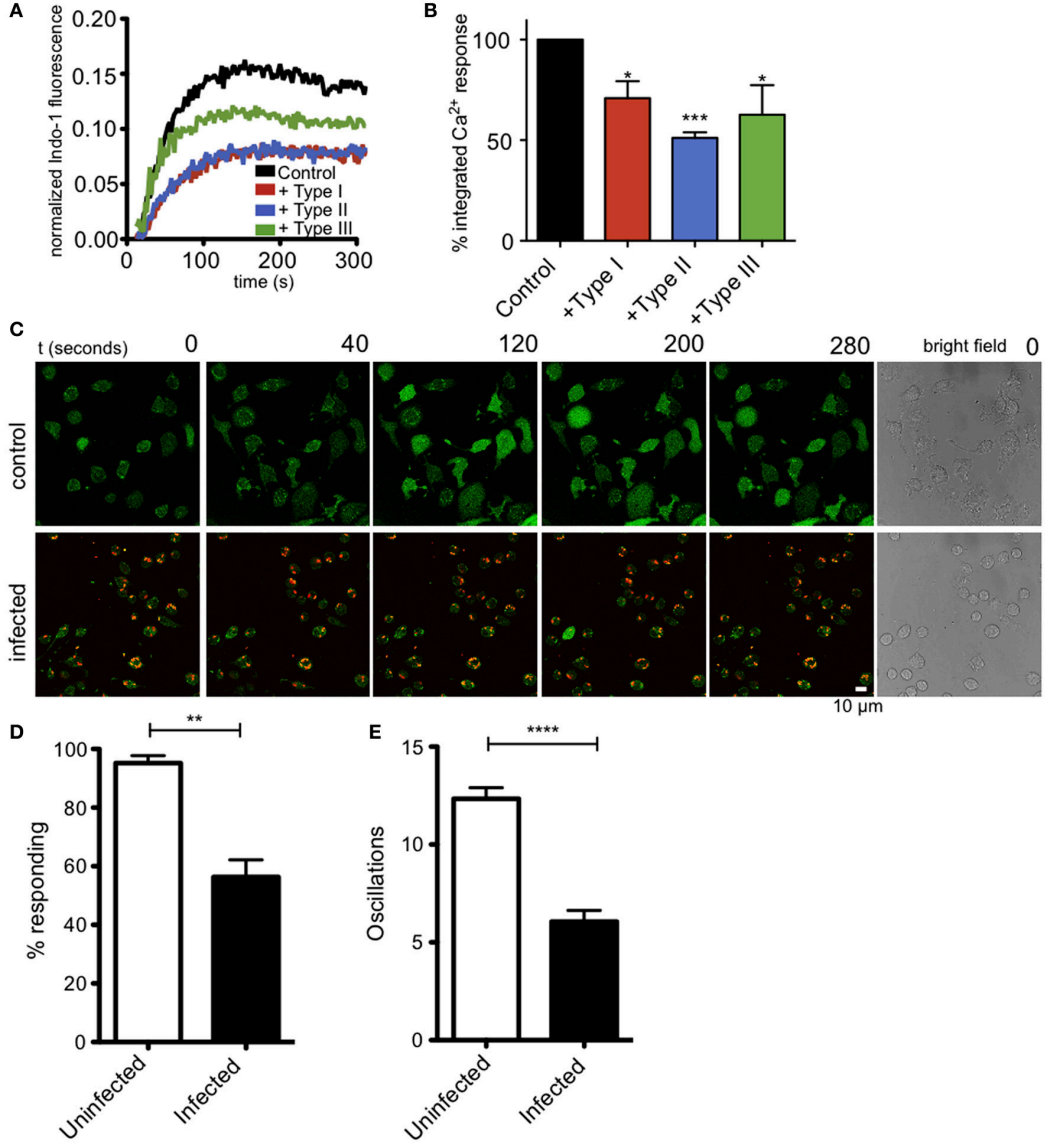
***T. gondii* infection reduces antigen-mediated Ca^2+^ responses in RBL mast cells. (A)** Representative indo-1 fluorescence measurements of Ca^2+^ responses to multivalent antigen (10 ng/ml DNP-BSA) in control uninfected (black), Type I-infected (red), Type II-infected (blue) or Type III-infected (green) infected cells (MOI 10:1). **(B)** Integrated Ca^2+^ responses over 300 s of stimulation as a percentage of control Ca^2+^ response. Histogram shows averages for 3–5 independent experiments. **(C)** Live Fluo-4 (green) Ca^2+^ imaging in uninfected or RH-tomato (red) infected RBL-cells. (see supporting movies M2 and M3). Quantification of the percentage of responding cells **(D)** or average number of Ca^2+^ oscillations per cell in 10 min **(E)** in live Ca^2+^ imaging experiments (*n* = 174 cells over 3 experiments). Error bars represent SEM (^*^P < 0.05, ^**^P < 0.01, ^***^P < 0.001, and ^****^P < 0.0001 relative to uninfected cells).

Ca^2+^ release from ER stores is mediated by binding of IP_3_ to its receptors in the ER membrane. This event triggers coupling of the ER-localized Ca^2+^ sensor, STIM1, and the plasma membrane Ca^2+^ channel Orai1, resulting in additional Ca^2+^ influx from extracellular space, a process known as store operated Ca^2+^ entry (SOCE). These events are important downstream steps in the pathway that leads to degranulation in mast cells (Di Capite and Parekh, 2009; Holowka et al., 2012). Measurement of intracellular Ca^2+^ in suspended RBL cells reveals that infection by *T. gondii* significantly reduces the Ca^2+^ response to antigen (Figure 3A). Integration of Ca^2+^ responses over 5 min shows a 39% reduction in Ca^2+^ mobilization in 3–5 independent experiments, averaged over all parasite types (Figure 3B).

In these bulk population measurements, it is difficult to evaluate what percent of infected cells are inhibited. We directly addressed this question by conducting live cell imaging experiments to evaluate Ca^2+^ responses in individual cells. Figure 3C, top panel, and movie M2 show that control, uninfected cells exhibit robust Ca^2+^ signaling in response to antigen, with 94% of the cells responding with an average of 12 oscillations during a 10-min period. In contrast, as shown in Figure 3C, bottom, and movie M3, the RH-tomato infected cells are much less responsive, such that only 58% of cells containing one or more parasites respond, typically with a slower onset and with an average of 6 oscillations over the same time period. These parameters are compared in Figures 3D,E.

**Figure 4:**
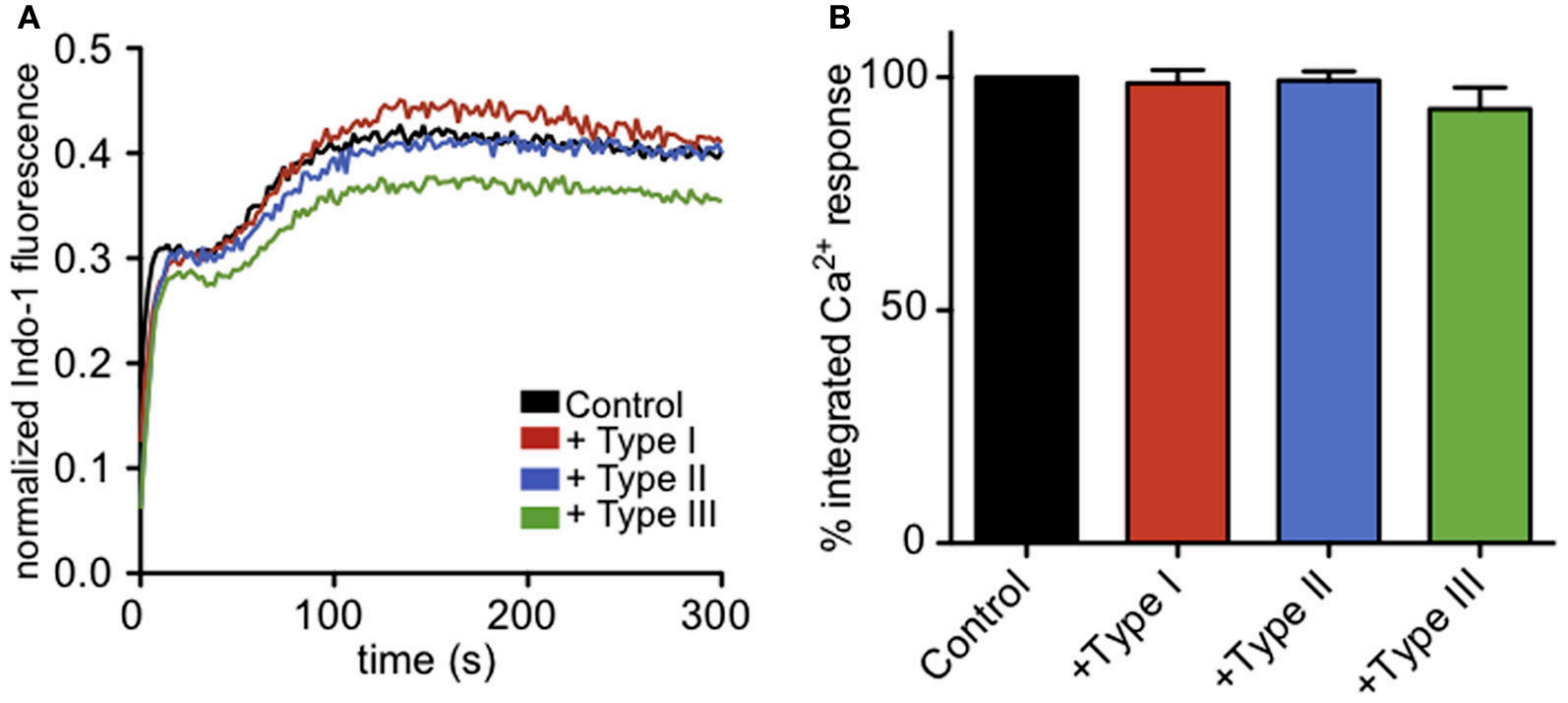
**Infection does not affect Ca^2+^ responses to ER store release by thapsigargin. (A)** Representative indo-1 fluorescence measurements of Ca^2+^ responses to 200 nM thapsigargin in control (black), Type I (red), Type II (blue), or Type III (green) infected cells (MOI 10:1). **(B)** Integrated Ca^2+^ responses over 300 s of stimulation as a percentage of control Ca^2+^ response. Bars show average of 3–5 independent experiments. Error bars represent SEM.

It is known that intracellular parasites interact extensively with the ER (Sinai and Joiner, 1997), and one possibility is that these interactions somehow block the exit of Ca^2+^ from the ER during antigen-stimulated, IP_3_-dependent depletion of ER Ca^2+^. Receptor-mediated, IP_3_-dependent release of Ca^2+^ from ER stores can be bypassed by treating cells with thapsigargin, a SERCA pump inhibitor, resulting in passive leakage of Ca^2+^ from the ER that activates SOCE. Under these conditions, cells infected with all types of *T. gondii* have Ca^2+^ responses comparable to uninfected cells (Figures 4A,B), showing that *T. gondii* is not directly blocking SOCE. These results contrast with those of Haroon and colleagues, who showed reduced thapsigargin-mediated Ca^2+^ responses in Toxoplasma-infected mouse neurons (Haroon et al., 2012). Collectively, our results indicate that a common factor, shared by the three genotypes of parasite, inhibits granule exocytosis via a mechanism that inhibits Ca^2+^ mobilization upstream of SOCE.

### PLCγ activation is reduced in *Toxoplasma*-infected RBL-2H3 cells

**Figure A1:**
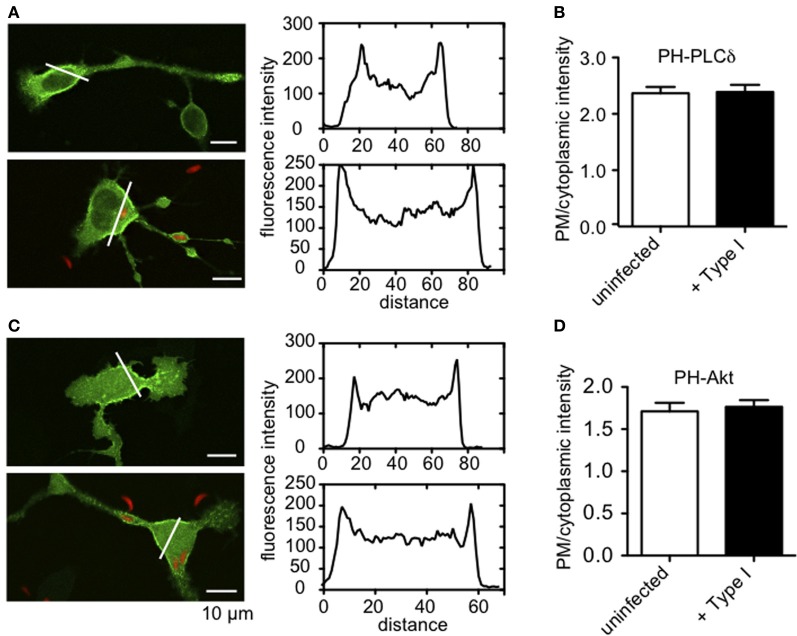
**PIP_2_ and PIP_3_ levels at the plasma membrane are not altered by *T. gondii* infection. (A)** Representative images showing uninfected (top) or RH-tomato infected (bottom) RBL cells transiently transfected with PH-PLCδ-EGFP. Accompanying fluorescence intensity profiles are taken along the white lines indicated in each image. **(B)** Ratio of plasma membrane to cytoplasmic PH-PLCδ-EGFP fluorescence [given by intensity profiles as shown in **(A)**] as a relative measure of PIP_2_ at the plasma membrane. **(C)** Representative images showing uninfected (top) or RH-tomato infected (bottom) RBL cells transiently transfected with PH-Akt-EGFP. Accompanying fluorescence intensity profiles are taken along the white lines indicated in each image. **(D)** Ratio of plasma membrane to cytoplasmic PH-Akt-EGFP fluorescence as a relative measure of PIP_3_ at the plasma membrane. Data in **(B)** and **(D)** represent at least 30 cells for each condition collected over 3 experiments. Error bars indicate SEM.

Antigen stimulation of FcεRI activates phospholipase Cγ (PLCγ), resulting in hydrolysis of PIP_2_ to DAG and IP_3_. This, in turn, triggers Ca^2+^ release from ER stores via IP_3_ receptors at the ER. One possible mechanism of inhibition of this process is that intracellular parasites might sequester PIP_2_, such that it is no longer available as a substrate for PLCγ. To address this possibility, we assessed whether infection by *Toxoplasma* alters the plasma membrane association of GFP-tagged PH-PLCδ that is highly specific for PIP_2_ (Ferguson et al., 1995). We found no appreciable differences in the abundance of PIP_2_ availability at the plasma membrane under these conditions (Figures A1A,B).

**Figure 5:**
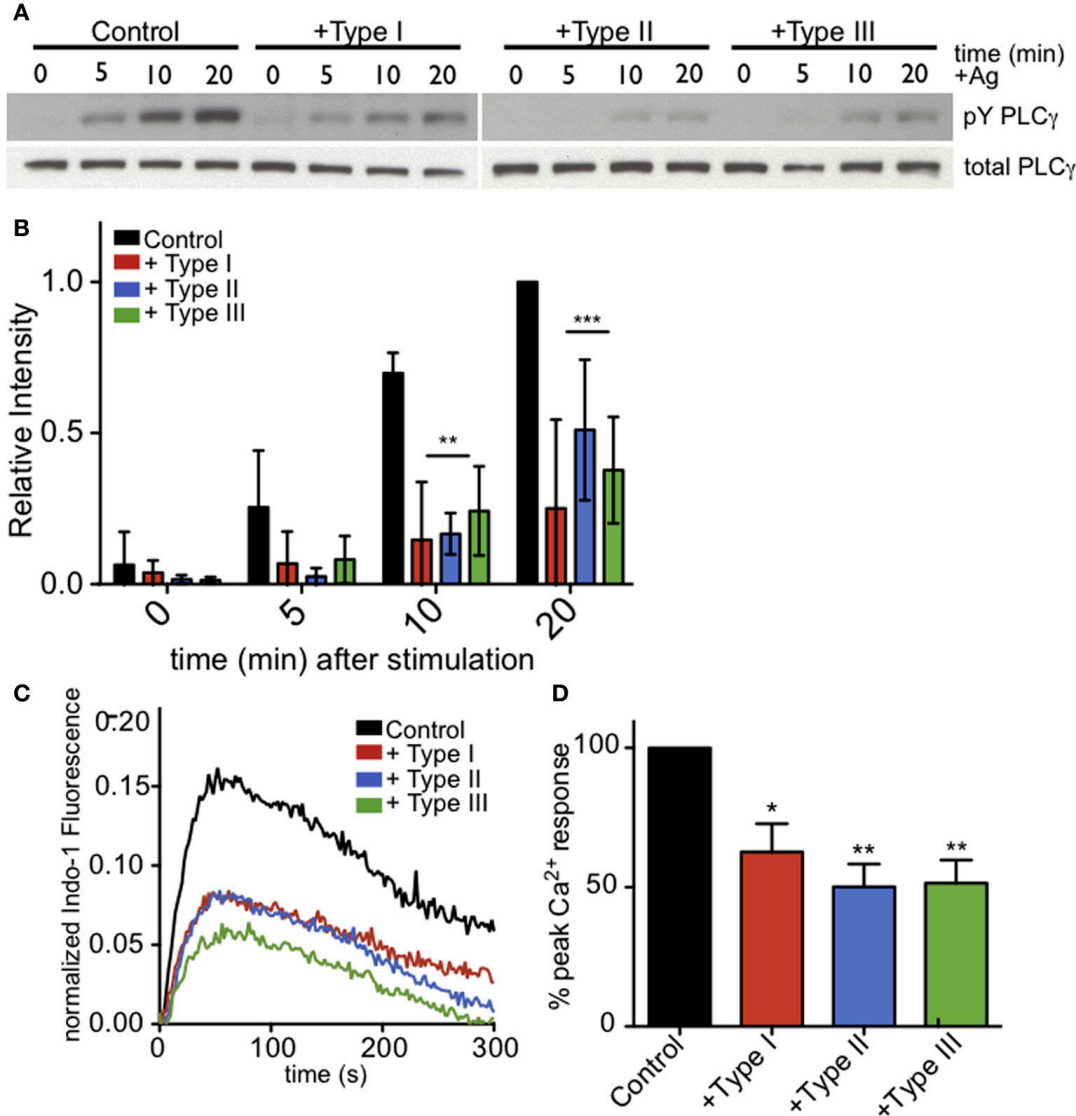
**PLCγ2 activity is reduced in *T. gondii*-infected RBL-2H3 cells. (A)** Representative western blot of phospho-PLCγ2 (top) or total PLCγ2 (bottom) in control or type I-,II- or III-infected (MOI 10:1, 1 h) cells lysed at 0, 5, 10, or 20 min after addition of multivalent antigen (10 ng/ml DNP-BSA). **(B)** Quantification of phospho-PLCγ2 analysis as shown in **(A)**. Error bars represent SD of 4 independent experiments (^**^P < 0.01,^***^P < 0.001, and ^****^P < 0.0001 relative to uninfected cells). **(C)** Representative indo-1 fluorescence measurements of Ca^2+^ responses to 10 ng/ml DNP-BSA in the absence of extracellular Ca^2+^ in control uninfected (black), Type I-infected (red), Type II-infected (blue) or Type III-infected (green) cells (MOI 10:1). **(D)** Peak Ca^2+^ responses in infected cells as a percentage of control Ca^2+^ response of uninfected cells in the absence of extracellular Ca^2+^. Histogram shows averages of 3 independent experiments. Error bars represent SEM (^*^P < 0.05 and ^**^P < 0.01 relative to control cells).

As phosphoinositide availability at the plasma membrane does not appear to be significantly changed in infected RBL cells, we next asked whether the parasite-mediated inhibition is due to a defect in PLCγ activation. For PLCγ to enzymatically cleave PIP_2_, it must be recruited to the plasma membrane and phosphorylated at specific tyrosine residues. Western blotting with anti-pY1217-PLCγ2 shows that antigen-stimulated phosphorylation at this residue is reduced in all infected samples (Figure 5A). At 10 min post-stimulation in the presence of types I, II and III parasites, Y1217-PLCγ2 phosphorylation is reduced by >65% in at least 3 independent experiments (Figure 5B). PLCγ1, like PLCγ2, shows reduced phosphorylation at its activating tyrosine, Y783 (N.L. Smith, data not shown).

To further assess the activity of PLCγ, we asked if antigen-mediated IP_3_ generation was reduced in *T. gondii* infected RBL cells. Specifically, we examined Ca^2+^ mobilization in the absence of extracellular Ca^2+^ as a measure of PLCγ-dependent Ca^2+^ release from ER stores. Figure 5C is a representative experiment that shows all parasite types significantly reduce the amount of Ca^2+^ released from ER stores in response to antigen. Over multiple experiments, parasite infection reduced the Ca^2+^ release from ER stores by 37, 50, and 49% for Types I, II, and III, respectively (Figure 5D).

### Syk kinase activity is reduced by *T. gondii* infection

**Figure 6:**
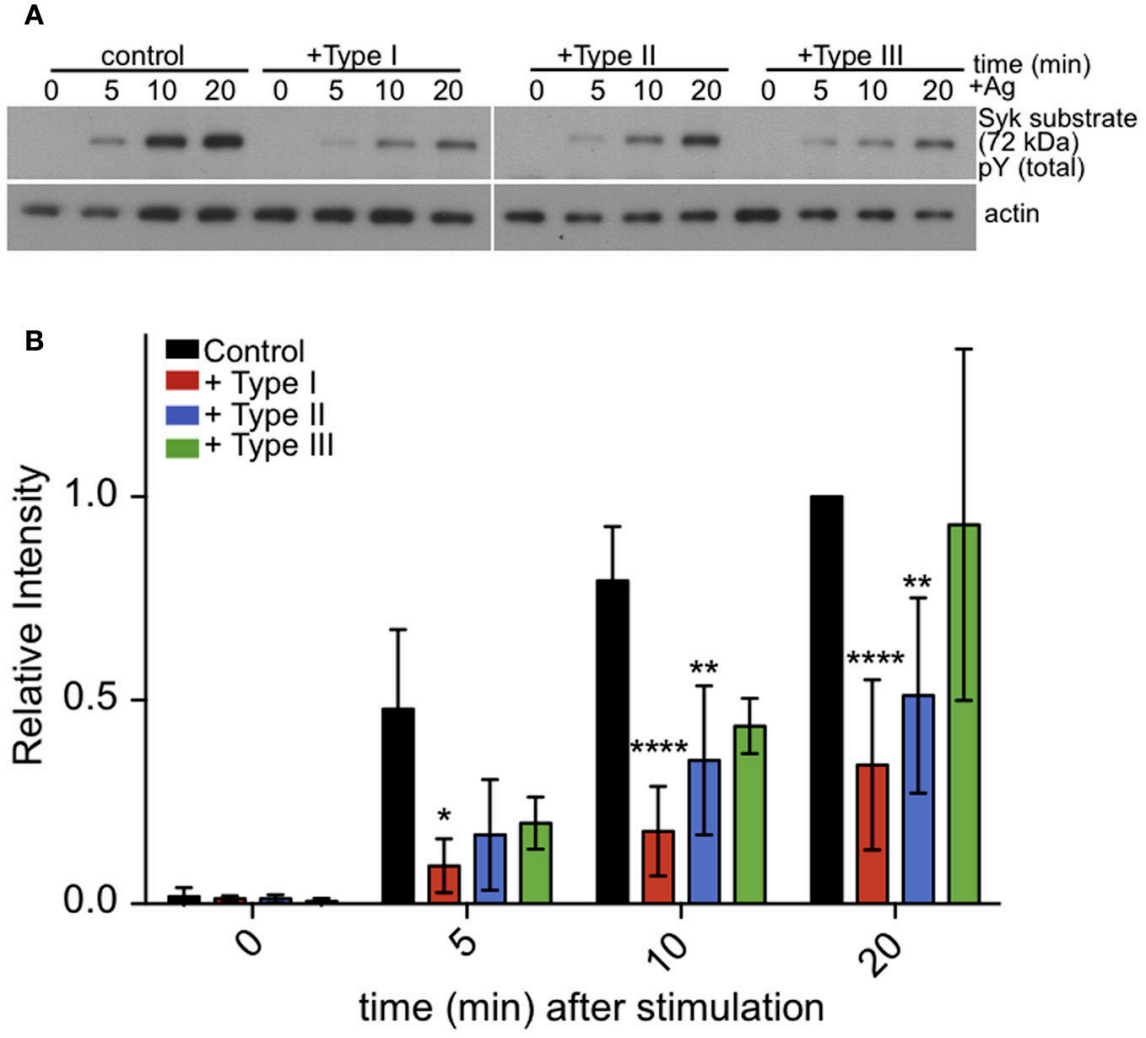
**Tyrosine phosphorylation of pp72 Syk substrate is reduced in *T. gondii* infected cells**. IgE-sensitized RBL cells were infected for 1 h with Type I, II or III tachyzoites as indicated. Antigen-stimulated cells were lysed at 0, 5, or 10 min after addition of multivalent antigen (10 ng/ml DNP-BSA). **(A)** Representative blot: Top panel shows phosphorylation of pp72 Syk substrate. Bottom panel shows loading control (α-tubulin) **(B)** Quantification of Syk substrate band intensity. Error bars represent SD of 4 independent experiments (^*^P < 0.05, ^**^P < 0.01, and ^****^P < 0.0001 relative to uninfected cells).

Following crosslinking of IgE/FcεRI complexes by multivalent antigen, tyrosine residues within ITAMs are phosphorylated in the cytoplasmic segments of the β and γ subunits of FcεRI. This, in turn, recruits and activates Syk tyrosine kinase. Syk kinase has a number of downstream targets, including PLCγ. Therefore, we asked if antigen stimulation of the phosphorylation of additional Syk substrates, detected as pp72 (Benhamou et al., 1993), is altered in infected cells. As we saw for PLCγ, stimulated phosphorylation of Syk substrate pp72 is also reduced in infected cells (Figures 6A,B). These results suggest that inhibition of PLCγ-mediated hydrolysis of PIP_2_ is due to reduction in the activation of Syk kinase.

### Inhibition of mast cell signaling by *T. gondii* requires parasite attachment, but not entry

**Figure 7:**
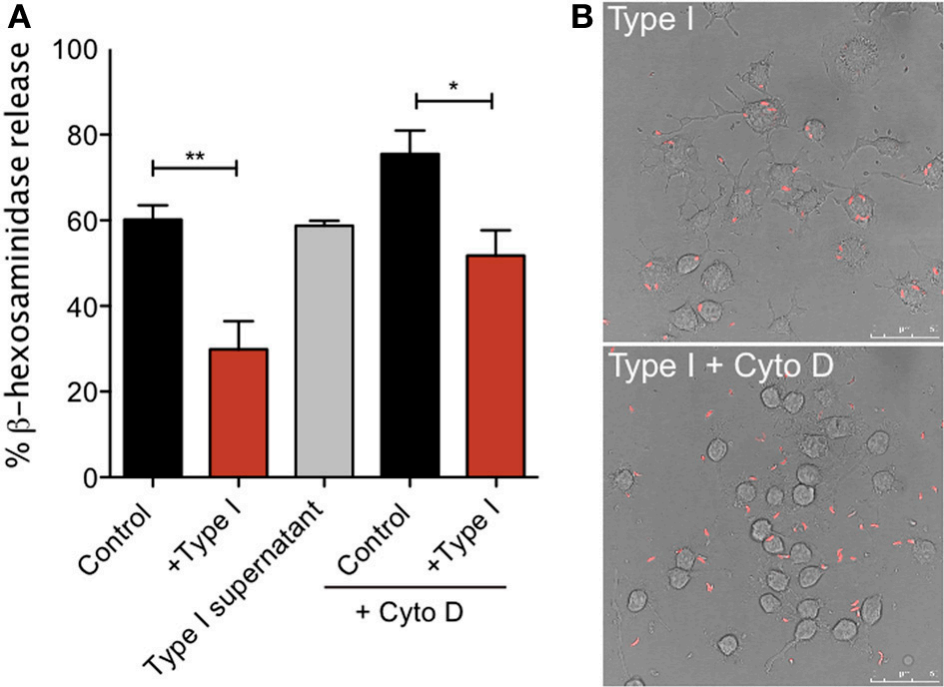
**Tachyzoite attachment, but not invasion, is necessary for inhibition of degranulation. (A)** RBL-2H3 cells were incubated with parasites (Type I) in the presence or absence of cytochalasin D or with supernatants from parasites (Type I supernatant). Degranulation stimulated by multivalent antigen (10 ng/ml DNP-BSA) was assessed by β-hexosaminindase release after 1 h of infection as described in Materials and Methods. For Control, + Type I, Control + Cyto D and + Type I + Cyto D error bars represent SEM (*n* = 4) (^*^P < 0.05 and ^**^P < 0.01 relative to control cells); for Type I supernatant, error bar represents range (*n* = 2). **(B)** Representative confocal microscopy of RBL cells infected with RH tomato parasites in the presence (bottom panel) or absence (top panel) of cytochalasin D.

Our single cell Ca^2+^ and degranulation measurements indicate that the inhibitory action of *T. gondii* requires direct contact between the host cell and parasite and possibly parasite entry. To more directly address these issues, we compared degranulation responses in RBL cells infected with the Type I parasites to responses to cells that were incubated with the supernatant from an equivalent number of parasites (Type I supernatant). As shown in Figure 7A, RH supernatant did not inhibit antigen-stimulated degranulation under conditions in which infection by intact parasites was effective (+ Type I vs. Type I supernatant). Additionally, degranulation is not inhibited by heat-killed parasites, fixed parasites or by supernatants from infected fibroblasts (N.L. Smith, data not shown).

Parasite entry depends primarily on the parasite actin cytoskeleton (Dobrowolski and Sibley, 1996; Hakansson et al., 2001). Therefore, infections carried out in the presence of the inhibitor of actin polymerization, cytochalasin D, result in a frustrated state where parasites attach and secrete proteins into the host cell but do not complete invasion (Hakansson et al., 2001). Cytochalasin D is known to enhance degranulation responses of RBL cells to antigen (Frigeri and Apgar, 1999), and, as expected, we see robust stimulated degranulation under these conditions (Figure 7A, Control + Cyto D). However, degranulation in the presence of cytochalasin D and parasites is still significantly reduced compared to cytochalasin D treatment alone (Figure 7A). The average inhibition over three independent experiments is 32%. Microscopic observations under these conditions confirmed that infection rates in the presence of cytochalasin D were extremely low: 4% in cytochalasin D treated samples compared to approximately 70% under control conditions (Figure 7B). These results indicate that inhibition under these conditions is due to attached, but not intracellular parasites. Although we cannot rule out the possibility that the attached parasite directly inhibits the host's tyrosine kinase activity without entering the cell, it is more likely that the agent responsible for mediating inhibition of mast cell signaling is secreted into the cell at the initiation of the invasion process.

## Discussion

Based on its global prevalence and its capacity to infect a multitude of hosts, *T. gondii* is regarded as one of the world's most successful parasites. It now appears that the success of this parasite is due, in part, to the arsenal of immune-modulatory mechanisms it employs. For example, this parasite blocks macrophage and dendritic cell responses to IFN-γ and LPS signaling. In part, this appears to be due to exploitation of STAT signaling as well as interference with chromatin remodeling (Leng and Denkers, 2009; Leng et al., 2009b). Our current study uncovers another mechanistic target of parasite interference, namely PLCγ-mediated Ca^2+^ mobilization.

*In vivo, T. gondii* is known to preferentially infect immune effector cells, including neutrophils, macrophages and dendritic cells (Denkers and Butcher, 2005; Bierly et al., 2008). Accordingly, we found that this infection suppresses receptor-mediated Ca^2+^ mobilization in murine neutrophils (Figure 1). To further characterize the mechanism of such effects, we chose the well-studied immune signaling model, FcεRI in mast cells, to address the molecular basis of *T. gondii* effects on acute immune signaling events that rely on Ca^2+^ signaling. FcεRI-triggered degranulation in mast cells begins within minutes of stimulation and continues over several tens of minutes. Intracellular Ca^2+^ levels are tightly regulated, and elevation of intracellular Ca^2+^ is critical in immune responses in multiple cell types and contexts (Andersson et al., 1986; Penner and Neher, 1988; Putney, 2009). Host Ca^2+^ responses in macrophages have been implicated in regulating the initial recognition of *T. gondii* that results in MAPK activity leading to IL-12 production (Masek et al., 2006). Furthermore, intracellular parasites are sensitive to host Ca^2+^ responses, and exogenous stimulation Ca^2+^ influx by treatment with Ca^2+^ ionophore triggers tachyzoite egress (Caldas et al., 2007).

Ca^2+^ mobilization is a central and well-studied aspect of IgE/FcεRI-mediated signaling in mast cells, including its role in granule exocytosis and cytokine production (Holowka et al., 2012). Our initial experiments revealed that parasite infection suppresses degranulation responses in RBL cells (Figure 2), and thus its inhibition of Ca^2+^ mobilization was a likely suspect. We showed that parasite infection blocks antigen-triggered, but not thapsigargin-triggered Ca^2+^ elevation (Figures 3, 4), indicating that infection acts upstream of Ca^2+^ release from stores. Inhibition of Ca^2+^ mobilization by *T. gondii* in the absence of extracellular Ca^2+^ further supports this explanation (Figure 5). Hydrolysis of PIP_2_ mediated by PLCγ to produce IP_3_ and DAG is critically important in Ca^2+^ mobilization by antigen in mast cells. One possible mechanism to account for decreased Ca^2+^ store release in response to antigen is that the parasite alters the amount or availability of PLCγ's substrate, PIP_2_, at the plasma membrane. However, our results are inconsistent with this explanation, as we detect no significant change in PIP_2_ levels at the plasma membrane due to infection by T. gondii. We do, however, observe a reduction in the level of activating tyrosine phosphorylation of PLCγ during infection (Figure 5), suggesting that *Toxoplasma* is reducing the host's capacity to hydrolyze PIP_2_.

**Figure 8:**
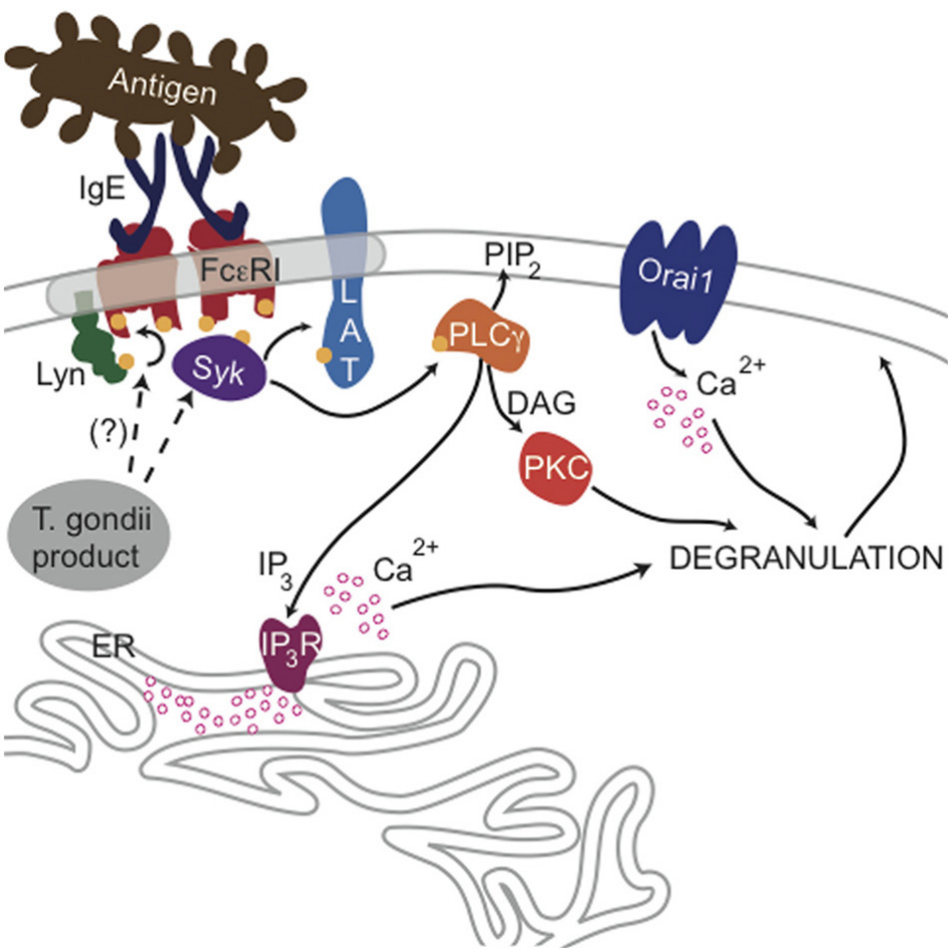
**Ca^2+^-mediated signaling through FcεRI is blocked by a *T. gondii*-secreted product**. Crosslinking of IgE/FcεRI complexes by multivalent antigen leads to phosphorylation of FcεRI subunits by Lyn kinase and subsequent recruitment and activation of Syk, which can then phosphorylate the adaptor protein LAT and other substrates. Consequent activation of PLCγ produces DAG and IP_3_, and IP_3_ activates IP_3_ receptors at the endoplasmic reticulum, causing Ca^2+^ release from ER stores and additional influx through Orai1 channels at the plasma membrane surface. Ca^2+^ mobilization and activation of PKC via DAG production is necessary for maximal degranulation of secretory lysosomes. The data presented here suggest that a secreted product of *T. gondii* blocks this signaling at the level of Syk phosphorylation leading to PLCγ activation.

PLCγ1 activity in mast cells is regulated by PI3-kinase-mediated production of PIP_3_ (Barker et al., 1998), but our results indicate that the parasite does not significantly change the level of PIP_3_ in infected cells (Figures A1C,D). Rather, our findings that phosphorylation of Syk substrates pp72, as well as PLCγ, are reduced in infected RBL cells, point to inhibition of the tyrosine kinase signaling cascade that culminates in PIP_2_ hydrolysis as the most immediate consequence of *T. gondii* infection. The earliest target in this cascade is not yet clear, as *T. gondii* infection caused some reduction in FcεRI ITAM phosphorylation by Lyn kinase that was not statistically significant (N.L. Smith, unpublished observations). Our data collectively suggest a model in which one or more *T. gondii*-derived proteins act directly to reduce the activity of PLCγ by inhibiting Syk activation, thereby reducing the levels of IP_3_ and inhibiting subsequent signaling steps (Figure 8).

Furthermore, our results suggest that a secreted product from *T. gondii* mediates this inhibition at an early step during the invasion process. Previous work determined that invasion is a multi-step process (Carruthers and Boothroyd, 2007), and, in early steps, parasites attach to the host plasma membrane and release the contents of the rhoptries into the cell (Hakansson et al., 2001). Quantitative trait locus analysis revealed that rhoptry proteins, including ROP16 and ROP18, which are secreted during invasion, are key virulence factors in *T. gondii* infection (Saeij et al., 2006; Taylor et al., 2006). Recently, ROP16 has been shown to directly phosphorylate STAT molecules (Yamamoto et al., 2009; Ong et al., 2010). However, lack of parasite strain specificity argues against a role for ROP16 and ROP18 in the effects reported here, as there are documented differences in the activity of these ROP proteins in the three types of parasites evaluated (Saeij et al., 2006; Taylor et al., 2006; Boyle et al., 2008). Furthermore, we tested ROP16 null parasites and found them equally capable of blocking Ca^2+^ responses (N.L. Smith, unpublished observations). Nevertheless, ROP protein early release, relation to virulence, and immunomodulatory capabilities make these proteins attractive candidates for the *Toxoplasma* secreted factor responsible for the reduction in Ca^2+^ mobilization (Ong et al., 2010; Butcher et al., 2011). Future work will focus on identifying which parasite-derived protein(s) are responsible for the inhibition we observe.

We also note that while our results indicate that *Toxoplasma* inhibits mast cell immune responses, this is not contradictory to reports that mast cell responses contribute to the primary host response to *Toxoplasma in vivo* (Ferreira et al., 2004; Sawesi et al., 2010). Our data show that PLCγ-mediated responses are reduced by Toxoplasma infection *in vitro*. Furthermore, our findings that PLCβ-mediated neutrophil responses are inhibited by *Toxoplasma* infection (Figure 1), as are voltage-gated neuronal Ca^2+^ responses (Haroon et al., 2012), indicate that there are likely multiple mechanisms employed by *T. gondii* to subvert normal Ca^2+^ signaling. In future studies it will be important to assess whether inhibition of Ca^2+^ signaling is manifest in other immune cells infected by *T. gondii*, as well as the mechanisms utilized. Collectively, our results indicate that *T. gondii* targets Syk-dependent PLCγ activation as one mechanism to interfere with immune signaling that depends on Ca^2+^ mobilization.

## Conflict of interest statement

The authors declare that the research was conducted in the absence of any commercial or financial relationships that could be construed as a potential conflict of interest.

## Supplementary material

Movie M1 **Live Imaging of RBL cell degranulation shows reduced responses in RH-tomato (red) infected cells compared to neighboring uninfected cells**. Images were acquired at a rate of 1.7 Hz. Multivalent antigen (10 ng/ml DNP-BSA) was added when indicated in movie and degranulation events are observed as FITC-dextran bursts (green). Movie plays at 10 × speed.

Movie M2 **Live Ca^2+^ imaging in uninfected RBL cells shows robust response to antigen**. RBL cells were loaded with fluo-4 (green) and imaged at a rate of 0.5 Hz. Multivalent antigen (10 ng/ml DNP-BSA) was added when indicated in movie. Movie plays at 12 × speed.

Movie M3 **Live Ca^2+^ imaging in RH-tomato (red) infected RBL cells show reduced response to antigen**. RBL cells were loaded with fluo-4 (green) and imaged at a rate of 0.5 Hz. Multivalent antigen (10 ng/ml DNP-BSA) was added when indicated in movie. Movie plays at 12 × speed.
